# Risk of dengue in Central Africa: Vector competence studies with *Aedes aegypti* and *Aedes albopictus* (Diptera: Culicidae) populations and dengue 2 virus

**DOI:** 10.1371/journal.pntd.0007985

**Published:** 2019-12-30

**Authors:** Basile Kamgang, Marie Vazeille, Armel N. Tedjou, Theodel A. Wilson-Bahun, Aurélie P. Yougang, Laurence Mousson, Charles S. Wondji, Anna-Bella Failloux

**Affiliations:** 1 Centre for Research in Infectious Diseases, Department of Medical Entomology, Yaoundé, Cameroon; 2 Institut Pasteur, Department of Virology, Unit of Arboviruses and Insect Vectors, Paris, France; 3 Department of Animal Biology, Faculty of Sciences, University of Yaoundé I, Yaoundé, Cameroon; 4 Faculty of Science and Technology, Marien Ngouabi University, Brazzaville, Congo; 5 Vector Biology Department, Liverpool School of Tropical Medicine, Liverpool, United Kingdom; Duke-NUS GMS, SINGAPORE

## Abstract

**Introduction:**

Dengue is the most important mosquito-borne diseases worldwide but was considered scarce in West-Central Africa. During the last decade, dengue outbreaks have increasingly been reported in urban foci in this region suggesting major epidemiological changes. However, in Central Africa where both vectors, *Aedes aegypti* and *Aedes albopictus* are well established, the role of each species in dengue transmission remains poorly investigated.

**Methodology/Principal findings:**

Field-collected strains of *Ae*. *aegypti* and *Ae*. *albopictus* from different ecological settings in Central Africa were experimentally challenged with dengue 2 virus (DENV-2). Mosquitoes were analysed at 14- and 21-days post-infection. Analysis provide evidence that both *Ae*. *aegypti* and *Ae*. *albopictus* in Central Africa were able to transmit dengue virus with *Ae*. *aegypti* exhibiting a higher transmission rate. Unexpectedly, two *Ae*. *aegypti* populations from Bénoué and Maroua, in northern Cameroon, were not able to transmit DENV-2.

**Conclusions/Significance:**

We conclude that both *Ae*. *aegypti* and *Ae*. *albopictus* are susceptible to DENV-2 and may intervene as active dengue vectors. These findings highlight the urgent need to plan a vector surveillance program and control methods against dengue vectors in Central Africa in order to prevent future outbreaks.

## Author summary

Dengue virus (DENV) is a flavivirus mainly transmitted to humans through the bite of infected mosquitoes notably *Aedes aegypti* and *Aedes albopictus*. In Central Africa where both vectors, *Ae*. *aegypti* and *Ae*. *albopictus* are well established, the role of each species in dengue transmission remains poorly investigated. Here, we assessed the vector competence of *Ae*. *aegypti* and *Ae*. *albopictus* collected in different ecological settings in Central Africa to transmit dengue 2 virus (DENV-2). We provide evidence that both *Ae*. *aegypti* and *Ae*. *albopictus* in Central Africa were able to transmit dengue virus with *Ae*. *aegypti* exhibiting a higher transmission rate. These findings could increase the risk of dengue outbreak in the region and emphasize the need for a comprehensive vector surveillance program to prevent and preparedness for an intervention in case of outbreaks.

## Introduction

Dengue is one of the most important arboviral diseases in the world with nearly 390 million annual dengue infections and 96 million (67–136 million) clinical cases [1]. Dengue is caused by a dengue virus (DENV) belonging to the genus *Flavivirus* (family *Flaviviridae*). There are four distinct, but closely related serotypes of dengue (DENV-1, DENV-2, DENV-3 and DENV-4). DENV is transmitted to humans through the bite of infected *Aedes* mosquitoes primarily *Aedes aegypti* Linneaus 1772 and *Aedes albopictus* (Skuse 1894).

In Africa, the situation of dengue was less critical as human cases were mainly associated with mild symptoms [2,3]. Haemorrhagic syndromes were only reported in East Africa [4,5]. However, dengue outbreaks have been reported recently in some West-Central African countries [6–10] suggesting a switch in the epidemiological dynamics of dengue. The two invasive species, *Ae*. *aegypti* and *Ae*. *albopictus* are well established in Africa. While *Ae*. *aegypti* native from Africa took 400–500 years to invade the tropical belt [11,12], *Ae*. *albopictus* originated from Asian forests has colonized all five continents in less than four decades [13,14]. *Aedes albopictus* has been first reported in Central Africa in early 2000s in Cameroon [15], and since then, this species has invaded almost all countries of the region including the Republic of Congo [16–18]. In sympatric areas, *Ae*. *albopictus* outcompetes with the native species *Ae*. *aegypti* [18–21]. Coincidentally, the emergence of arboviral diseases such as dengue and chikungunya in Central Africa has coincided with the establishment of *Ae*. *albopictus* in this region. Indeed, *Ae*. *albopictus* was identified as the main vector during concurrent dengue/chikungunya outbreak in Gabon in 2007 [8,22], and in Cameroon in 2006 [23]. During the last two decades, DENV-1 and DENV-2 mainly, were circulating in Cameroon [24–29]. Nationwide surveillance of dengue in 2006/2007 only revealed that seroprevalence (IgG and IgM antibodies) was higher in Douala [29]. In the neighbouring country, the Republic of the Congo, only little information is known about dengue circulation. The vector competence (which refers to the potential of an arthropod to ingest the pathogen, ensure replication, dissemination and transmission) which is one of the main factors required to establish the epidemiological role of mosquitoes in transmission is poorly studied in Central Africa. Previous studies only focused on infection and dissemination rates [8,30,31] and not transmission potential (i.e. virus detection in mosquito saliva). To fill this important gap, we performed a comparative analysis aiming to assess the ability of *Ae*. *aegypti* and *Ae*. *albopictus* collected in different ecological settings in Central Africa to transmit DENV-2.

## Materials and methods

### Ethics statement

This study was approved by the Cameroonian national ethics committee for human health research N°2017/05/911/CE/CNERSH/SP. Oral consent to inspect the potential breeding sites was obtained in the field in household or garage owners. The Institut Pasteur animal facility has received accreditation from the French Ministry of Agriculture to perform experiments on live animals in compliance with the French and European regulations on care and protection of laboratory animals (EC Directive 2010/63, French Law 2013–118, February 6th, 2013). All experiments were approved by the Ethics Committee and registered under the reference APAFIS6573-201606l412077987 v2.

### Mosquito sampling

**Fig 1:**
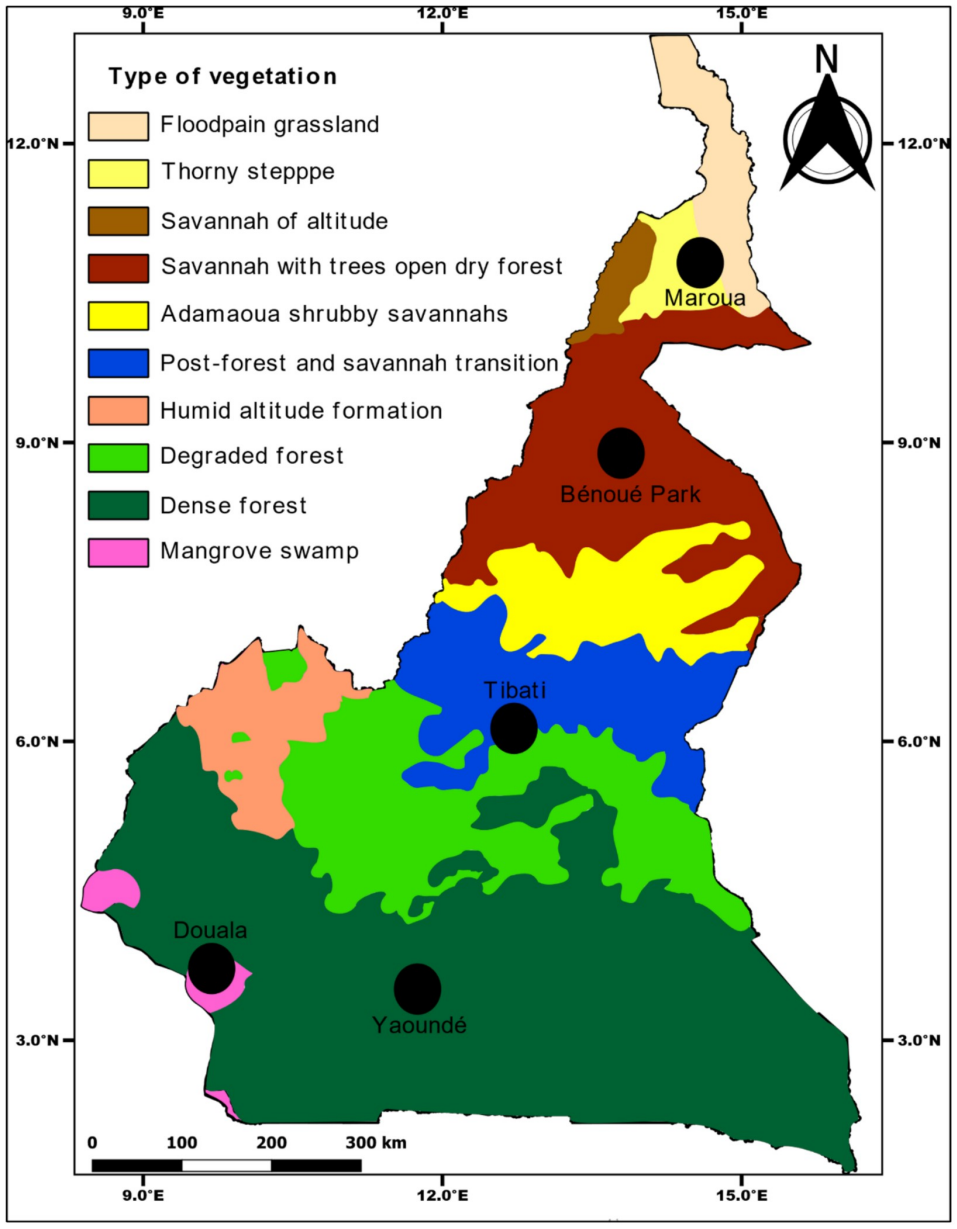
Map of Cameroon vegetation showing the sampling sites.

Larvae and pupae were collected from August 2017 to April 2018 in several locations in Central Africa including Brazzaville (Republic of the Congo), Yaoundé, Douala, Tibati and Bénoué National Park (Cameroon, Fig 1). Each of these locations have been previously characterised [18,19]. In each location, mosquitoes were collected in peri-urban (i.e. peripheral area of the city) and downtown (i.e. city centre with high building density) from a minimum of 20 containers per environment. Immature stages of *Aedes* were transported in the insectary and pooled together according to the city. Larvae were raised until adults and identified morphologically. Adults from same location and species were reared at 28°±1°C under 12h dark:12h light cycle and 80% relative humidity. Eggs obtained (Table 1) were transported to the Institut Pasteur Paris, reared to adult stage and used to challenge with DENV-2.

**Table 1:** Origin of *Ae*. *aegypti* and *Ae*. *albopictus* used for vector competence.

Location	Species	Generation
Yaoundé urban	*Ae*. *albopictus*	G2
Tibati	*Ae*. *albopictus*	G2
Douala	*Ae*. *albopictus*	G2
Brazzaville	*Ae*. *albopictus*	G5
Yaoundé urban	*Ae*. *aegypti*	G2
Yaoundé rural	*Ae*. *aegypti*	G2
Bénoué Parc	*Ae*. *aegypti*	G4
Brazzaville	*Ae*. *aegypti*	G2
Maroua	*Ae*. *aegypti*	G2
Douala	*Ae*. *aegypti*	G2

### Virus strain

The dengue 2 virus (DENV-2) strain provided by Leon Rosen (Institut Pasteur, Paris, France) was isolated in 1974 from a human sera from Bangkok, Thailand [32]. This virus had been passed only in different mosquito species (*Toxorhynchites amboinensis*, *Ae*. *albopictus*, and *Ae*. *aegypti*) by intrathoracic inoculation. Viral stocks were produced by inoculating *Ae*. *albopictus* cells (C6/36 clone) with triturated infected mosquitoes.

### Challenging mosquitoes with DENV-2

For each sample, six batches of 60 7–10 day old females were challenged with an infectious blood meal (Institut Pasteur, Paris, France) containing 1.4 mL of washed rabbit erythrocytes and 700 μL of viral suspension. The blood meal was supplemented with adenosine 5’-triphosphate (ATP) as a phagostimulant at a final concentration of 1 mM and provided to mosquitoes at a viral titre of 10^7^ focus-forming unit (ffu)/mL using a Hemotek membrane feeding system (Hemotek Ltd, Blackburn, United Kingdom). Mosquitoes were allowed to feed for 20 min through a piece of pork intestine (Institut Pasteur, Paris, France) covering the base of a Hemotek feeder maintained at 37°C. Fully engorged females were transferred in cardboard containers and maintained with 10% sucrose under controlled conditions (28±1°C, relative humidity of 80%, light: dark cycle of 12 h: 12 h) for up to 21 days with mosquito analysis 14 and 21 days post-infection (dpi). 21–32 mosquitoes were examined at each dpi.

### Infection, dissemination and transmission assays

For each mosquito examined, body (abdomen and thorax) and head were tested respectively for infection and dissemination rates at 14 and 21 dpi. For this, each part was ground individually in 300 μL of L15 medium (Invitrogen, CA, USA) supplemented with 2% fetal serum bovine (FBS), and centrifuged at 10,000×g for 5 min at +4°C. The supernatant was processed for viral titration. Saliva was collected from individual mosquitoes using technique of forced salivation as described previously [33]. Briefly, mosquitoes were cool anesthetized, wings and legs of each mosquito were removed and the proboscis inserted into a tip of 20 μL containing 5 μL of FBS. After 30 min, FBS containing saliva was mixed in 45 μL of L15 medium for titration.

Infection rate (IR) refers to the proportion of mosquitoes with infected body (i.e. abdomen and thorax) among tested mosquitoes. Disseminated infection rate (DIR) corresponds to the proportion of mosquitoes with infected head among the previously detected infected mosquitoes (i.e. virus positive abdomen/thorax). Transmission rate (TR) represents the proportion of mosquitoes with infectious saliva among mosquitoes with disseminated infection. Vector competence can be summarized by the transmission efficiency (TE) which was calculated as the proportion of mosquitoes with infectious saliva among total of mosquitoes tested [34].

### Viral titration by focus forming assay

Samples were titrated by focus fluorescent assay on *Ae*. *albopictus* C6/36 cells [35]. Body, head and saliva suspensions were serially diluted in L15 medium supplemented with 2% of FBS and inoculated onto cells in 96-well plates. After incubation of 5 days at 28°C, samples were fixed with 0.1mL/well of formaldehyde 3.6% in phosphate buffer saline (PBS) during 20 min at room temperature. Then, plates were stained using antibodies specific to DENV as the primary antibody, and conjugated goat anti-mouse immunoglobulin G (Alexa Fluor 488) as the second antibody (Life Technologies, CA, USA). Titres were expressed as ffu/mL.

### Statistical analysis

All statistical analyses were performed with R software v 3.5.2 (R Core Team, Vienna, Austria). Qualitative variables were expressed as proportion and compared using Fisher’s exact test (RVAideMemoire package). While quantitative variables were described as mean and compared using non-parametric test of Kruskal-Wallis because of non-normal distribution. Pairwise comparison were performed using Fisher’s exact test for proportions and Kruskal-wallis test for means. *P-value* <0.05 was considered as statistically different.

## Results

### Infection and disseminated infection rates in *Ae*. *albopictus* and *Ae*. *aegypti*

**Fig 2:**
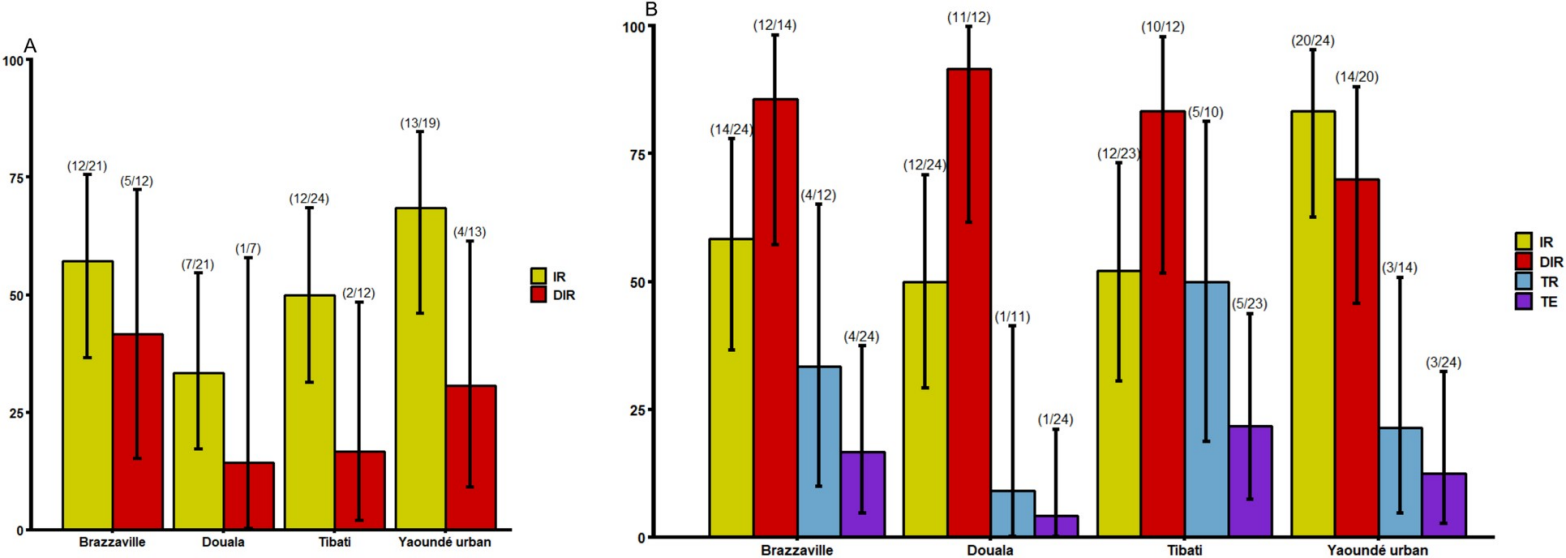
Infection, disseminated infection, transmission rates and transmission efficiency of *Ae*. *albopictus* from Central Africa to dengue virus. A) Infection and disseminated infection rates at 14 days post-infection (dpi). B) Infection, disseminated infection, transmission rates and transmission efficiency at 21 dpi. Error bars show the 95% confidence interval. In brackets, the number of mosquitoes examined. IR: the proportion of mosquitoes with infected body among engorged mosquitoes; DIR: the proportion of mosquitoes with infected head among mosquitoes with infected body; TR: the proportion of mosquitoes with infectious saliva among mosquitoes with infected head. TE: the proportion of mosquitoes with infectious saliva among all analysed ones.

**Fig 3:**
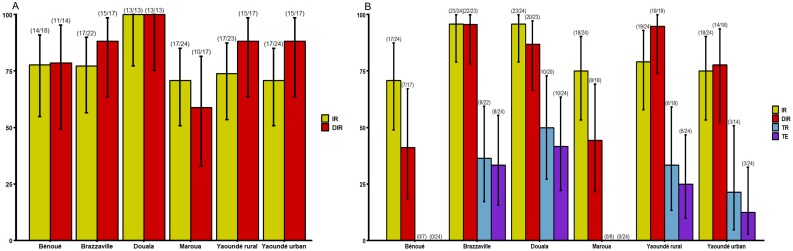
Infection, disseminated infection, transmission rates and transmission efficiency of *Ae*. *aegypti* from Central Africa to dengue virus. A) Infection and disseminated infection rates at 14 days post-infection (dpi). B) Infection, disseminated infection, transmission rates and transmission efficiency at 21 dpi. Error bars show the 95% confidence interval. In brackets, the number of mosquitoes examined. IR: the proportion of mosquitoes with infected body among engorged mosquitoes; DIR: the proportion of mosquitoes with infected head among mosquitoes with infected body; TR: the proportion of mosquitoes with infectious saliva among mosquitoes with infected head. TE: the proportion of mosquitoes with infectious saliva among all analysed ones.

To determine if *Ae*. *aegypti* (six populations) or *Ae*. *albopictus* (four populations) were more likely to sustain DENV outbreak in Central Africa, the ability of the virus to replicate and disseminate in both species was examined at 14 and 21 dpi as well as DENV particles excreted in saliva (only at 21 dpi) (Figs 2 and 3). At 14 dpi, *Ae*. *albopictus* infection rate (IR) ranged from 33.3% in Douala population to 68.4% in Yaoundé urban population but no statistical difference was detected (Fig 2A, Fisher’s Exact test: *P* = 0.16). For DIRs, similar trend was observed with lowest rate in Douala population (14.3%) and highest in Brazzaville population (41.6%) (Fig 2A, Fisher’s Exact test: *P* = 0.47). While for *Ae*. *aegypti*, results exhibited higher IRs ranging from 70.83% for Maroua to 100% for Douala populations and DIRs varying from 58.82% for Maroua to 100% for Douala populations. When considering all populations of same species, IRs for *Ae*. *aegypti* (mean = 76.61%) was significantly higher than for *Ae*. *albopictus* (mean = 51.76%) (Fisher’s exact test: *P* = 0.0003). Similar pattern was found for DIRs (*Ae*. *aegypti*: mean = 83.15% and *Ae*. *albopictus*: mean = 27.27%) (Fig 3A, Fisher’s exact test: *P*<10^−6^).

At 21 dpi, *Ae*. *albopictus* displayed higher IRs ranging from 50% for Douala population to 83.3% for Yaoundé urban and were not significantly different (Fisher exact test: *P* = 0.06). But pairwise comparisons showed that significant difference was found between Douala and Yaoundé urban (Fisher’s exact test: *P* = 0.03), Tibati and Yaoundé urban (Fisher’s exact test: *P* = 0.03). Higher DIRs was also reported: it varied from 70% for Yaoundé urban population to 91.66% for Douala population but no significant difference was found according to population (Fisher’s exact test: *P* = 0.52). For *Ae*. *aegypti*, IRs ranged from 70.83% for Bénoué population to 95.83% for Brazzaville and Douala populations and were not statistically different (Fisher exact test: *P* = 0.06). In contrast, a higher significant variation of DIRs was reported: it ranged from 41.17% for Bénoué population to 95.65% for Brazzaville population (Fisher exact test: *P*<10^−6^). Overall, IRs for *Ae*. *aegypti* (mean = 81.94%) were significantly higher than for *Ae*. *albopictus* (mean = 61.05%) (Fisher exact test: *P* = 0.0005). For DIR, no significant difference was found between *Ae*. *aegypti* and *Ae*. *albopictus* population (Fisher exact test: *P* = 0.45).

### Transmission rate and efficiency

**Fig 4:**
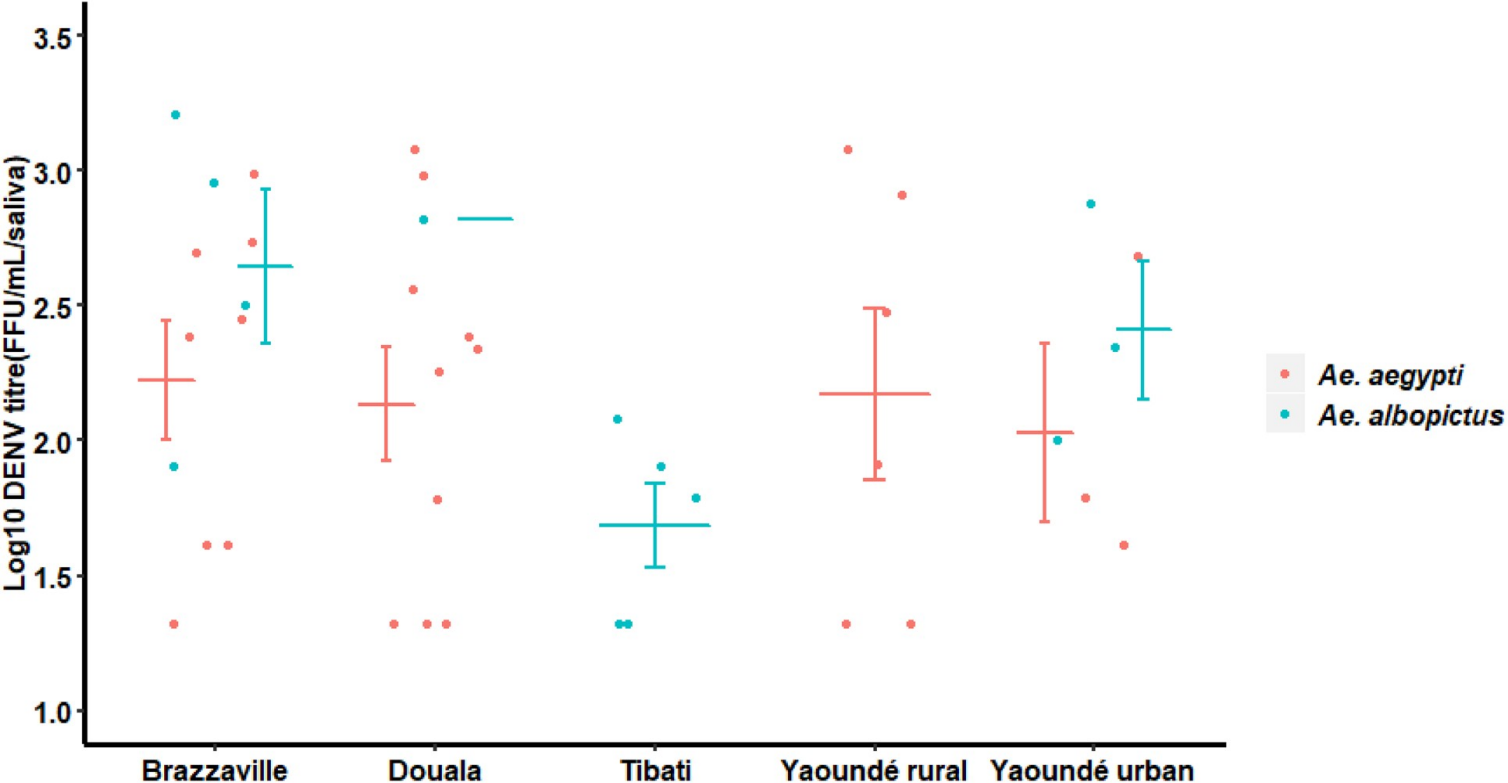
Dengue virus titres in saliva of *Ae*. *aegypti* and *Ae*. *albopictus* at 21 days post-infection. The bars indicate the confidence interval of the mean for viral load in each population.

Transmission rate (TR) and Transmission efficiency (TE) were assessed at 21 dpi in four *Ae*. *albopictus* and six *Ae*. *aegypti* populations (Figs 2B and 3B). In *Ae*. *albopictus*, DENV was detected in saliva of four populations with TRs ranging from 9.1% (1/11) for Douala to 50% (5/10) for Tibati populations; TRs were not statistically different (Fig 2B, Fisher exact test: *P* = 0.2). In contrast, for *Ae*. *aegypti*, DENV was not detected in saliva of Maroua and Bénoué populations, both located in northern Cameroon suggesting a low vector competence of these populations. For the other *Ae*. *aegypti* populations, TR ranged from 21.42% for Yaoundé urban population to 50% for Douala population (Fig 3B, Fisher exact test: *P* = 0.4). Overall, no significant difference was reported among *Ae*. *aegypti* and *Ae*. *albopictus* regarding TRs and TEs (Fisher exact test: *P*>0.05). When comparing populations from sympatric areas, TRs were significantly higher for *Ae*. *aegypti* (mean = 50%) than for *Ae*. *albopictus* (mean = 27.7%) (Fisher exact test: *P* = 0.007) while for viral load, no significant difference was reported between both species (Chi-squared = 0.14, df = 1, *P* = 0.70). For *Ae*. *aegypti*, no significant variation of viral loads was reported according to population (Fig 4; Chi-squared = 0.29, df = 3, *P* = 0.96) while for *Ae*. *albopictus*, a significant difference of viral loads was detected between Tibati and Brazzaville samples (Fig 4; Chi-squared = 2.31, df = 1, *P* = 0.018).

## Discussion

During the past decade, there has been a rise of dengue cases in urban foci in Central Africa notably in Cameroon [26–29]. Even suspected, vectors were not well identified and characterised. In this study, we assessed for the first time, the ability of *Ae*. *aegypti* and *Ae*. *albopictus* collected in different ecological settings (Fig 1) in Central Africa to transmit DENV-2, a serotype repeatedly reported in the region [9,29]. We demonstrated that DENV-2 was able to replicate, disseminate and be secreted in saliva of both *Ae*. *aegypti* and *Ae*. *albopictus* populations from Central Africa, thus enable to transmit DENV. However, infection rates were significantly higher for *Ae*. *aegypti* than for *Ae*. *albopictus* at 14 and 21 dpi. Disseminated infection rates followed the same trend at 14 dpi. Nevertheless, DENV was detected in saliva of all *Ae*. *albopictus* populations tested while for *Ae*. *aegypti*, virus was not detected in both populations (2/6) from northern Cameroon, Bénoué and Maroua. These results suggest that vector competence of *Ae*. *aegypti* to DENV-2 in Central Africa vary significantly according to geographical population as previously suggested elsewhere [36,37]. This may due to the fact that populations from Bénoué and Maroua exhibited an extrinsic incubation period longer than 21 days; to note, the extrinsic incubation period refers to the duration between the ingestion of an infectious blood meal and the excretion of virus in saliva when the mosquito bites [38]. It depends on the three-way combination of mosquito, virus and environment described under genotype-by-genotype-by-environment (GxGxE) interactions [39]. In addition, low vector competence in these populations would be due to presence of specific refractory genes [40,41]. Indeed, refractoriness of mosquito to dengue virus may be caused by different parameters like microbiome composition as bacterial symbionts of mosquitoes have been shown to alter the vector competence to arboviruses [42] and immune system of mosquito since it was demonstrated that anti-viral immunity in mosquito vectors is critical to prevent virus replication and transmission [43]. Further investigations in this regard are needed to elucidate.

Moreover, the seroprevalence of dengue examined in 2006/2007 in three main cities of Cameroon located in different ecological settings revealed that anti-DENV IgG and IgM antibodies varied significantly with a higher prevalence reported in Douala [29], location where the highest transmission rate and viral load were also detected in *Ae*. *aegypti* in this study. Beside the mosquito genetic background, mosquito microbiome can modulate arbovirus transmission [42,44,45]. The transmission rate was higher for *Ae*. *aegypti* compared to *Ae*. *albopictus* in locations where both species are sympatric. This result is in agreement with the fact that *Ae*. *aegypti* is considered as a major dengue vector, and *Ae*. *albopictus*, the secondary one [46]. Meanwhile, it would be interesting to highlight that *Ae*. *albopictus* can become a major dengue vector in the absence of *Ae*. *aegypti* as reported previously in China, the Seychelles, Japan, Hawaii and on La Réunion [47] or when *Ae*. *albopictus* becomes the most prevalent species as reported in Gabon [8]. Nevertheless, infection and disseminated infection rates assessed for *Ae*. *albopictus* in this study are similar to those reported in previous studies in Africa [8,31] and in Southeast Asia [48]. For *Ae*. *aegypti*, infection and disseminated infection rates are higher compared to that previously reported in Cameroon (17.2% to 59.7%) but similar to that often reported outside Africa [37,48]. Albeit *Ae*. *aegypti* is more competent than *Ae*. *albopictus* to transmit DENV, some parameters can influence DENV transmission in nature, such as vector densities, host preference, virus evolution and proportion of immunologically naive people [49]. Additional studies using a local strain of DENV circulating in Central Africa are needed to validate these results. Regarding vector densities, recent studies in Cameroon and Republic of Congo revealed that *Ae*. *albopictus* tends to replace *Ae*. *aegypti* in most areas where both species are sympatric [18,19]. It was also demonstrated that in Yaoundé (Cameroon) *Ae*. *albopictus* preferentially fed on humans rather than on available domestic animals [50] Data generated in our study demonstrated that both *Ae*. *aegypti* and *Ae*. *albopictus* can sustain dengue transmission in Central Africa. This could increase the risk of dengue outbreak in the region and urge the need of a vector surveillance program to prevent and preparedness for an intervention in case of outbreaks.
